# The use of everolimus to target carcinogenic pathways in a patient with renal cell carcinoma and tuberous sclerosis complex: a case report

**DOI:** 10.1186/1752-1947-8-95

**Published:** 2014-03-10

**Authors:** Hye Sook Kim, Seung Tae Kim, Seok Ho Kang, Deuk Jae Sung, Chul Hwan Kim, Sang Won Shin, Yeul Hong Kim, Won Yong Cho, Kyong Hwa Park

**Affiliations:** 1 Division of Oncology/Hematology, Department of Internal Medicine, College of Medicine, Korea University, Inchon-ro 73, Seongbuk-Gu, Seoul 136-702, Korea; 2 Department of Urology, College of Medicine, Korea University, Inchon-ro 73, Seongbuk-GuSeoul 136-702, Korea; 3 Department of Radiology, College of Medicine, Korea University, Inchon-ro 73, Seongbuk-Gu, Seoul 136-702, Korea; 4 Department of Pathology, College of Medicine, Korea University, Inchon-ro 73, Seongbuk-Gu, Seoul 136-702, Korea; 5 Division of Nephrology, Department of Internal Medicine, College of Medicine, Korea University, Inchon-ro 73, Seongbuk-Gu, Seoul 136-702, Korea

**Keywords:** Everolimus, Mammalian target of rapamycin inhibitor, Renal cell carcinoma, Tuberous sclerosis complex

## Abstract

**Introduction:**

An increased understanding of the genetic pathways involved in renal cell carcinoma has resulted in the development of various drugs that target relevant signaling cascades for the specific treatment of this disease. However, no validated predictive markers have been identified to guide the decision whether patients should receive vascular endothelial growth factor–targeted therapy or mammalian target of rapamycin–targeted therapy. We present what is, to the best of our knowledge, the first case of renal cell carcinoma in a patient with tuberous sclerosis complex who was successfully treated with everolimus.

**Case presentation:**

The patient was a 49-year-old Korean woman with tuberous sclerosis complex and recurrent renal cell carcinoma. The patient was treated with the tyrosine kinase inhibitor sunitinib followed by the mammalian target of rapamycin inhibitor everolimus. This treatment resulted in a prolonged response and significant clinical benefit. Notably, everolimus ameliorated the symptoms related not only to renal cell carcinoma but also to tuberous sclerosis complex.

**Conclusion:**

This case provides a rationale for the use of everolimus as first-line treatment for this specific patient population in order to target the correct pathway involved in carcinogenesis.

## Introduction

Tuberous sclerosis complex (TSC) is an autosomal dominant disease that was first described by Bourneville in 1880 [1]. TSC is characterized by hamartomatous lesions in various organs, such as the skin, retina, kidney, central nervous system, heart and lungs [1]. TSC arises from genetic alterations in one of two genes: *TSC1* (encoding hamartin) or *TSC2* (encoding tuberin). Tumor cells taken from patients with TSC have been shown to exhibit active mammalian target of the rapamycin (mTOR) signaling, so mTOR inhibitors have been identified as potential therapeutic agents.

In this report, we describe the case of a patient with TSC in whom TSC-related symptoms improved and a partial response to recurrent renal cell carcinoma (RCC) was achieved after treatment with the mTOR inhibitor everolimus. The findings of this case suggest that everolimus is an effective and appropriate treatment for patients with RCC related to TSC.

## Case presentation

**Figure 1:**
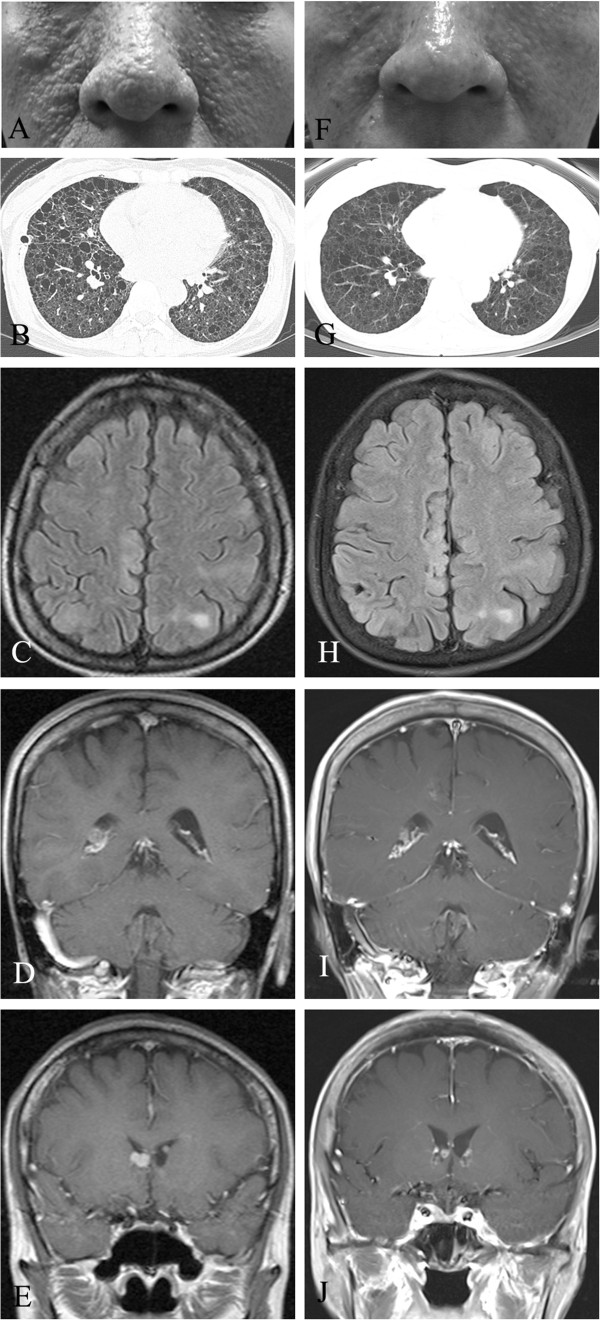
**Clinical features of tuberous sclerosis complex at diagnosis and after treatment with everolimus. (A)** Photograph of the patient’s facial fibroadenoma. **(B)** Chest computed tomographic scan of multiple cystic lesions consistent with lymphangioleiomyomatosis. **(C)** through **(J)** Brain magnetic resonance imaging scans of the patient. **(C)** Cortical tubers. **(D)** Multiple subependymal nodules. **(E)** Subependymal giant cell astrocytoma. **(F)** through **(J)** Changes after treatment with everolimus.

In May 2009, a 49-year-old Korean woman presented with complaints of fever and left flank pain to our department. For the previous two decades, she had received regular medical care for TSC, which had been diagnosed on the basis of angiomyolipomas (AMLs) in the kidney and fibroadenoma on the face (Figure 1A). Her medical history included multiple episodes of seizures during infancy with no subsequent evidence of significant intellectual deficit. At the age of 40, the patient experienced spontaneous pneumothorax in her right lung. Chest computed tomography (CT) revealed multiple cystic lesions consistent with lymphangioleiomyomatosis (LAM) (Figure 1B). Additional systemic evaluations for clinical TSC manifestations revealed several cortical tubers (Figure 1C), multiple subependymal nodules (Figure 1D), a subependymal giant cell astrocytoma (SEGA) detected on brain magnetic resonance images (Figure 1E) and retinal hamartomas identified during ophthalmologic examinations. Echocardiography and endoscopy of the stomach and colon revealed no abnormal findings. The aforementioned clinical findings fulfill the four major diagnostic criteria for a diagnosis of TSC; thus the diagnosis was confirmed without genomic analysis. The patient’s family history was unremarkable before her daughter developed facial angiofibromatosis.

**Figure 2:**
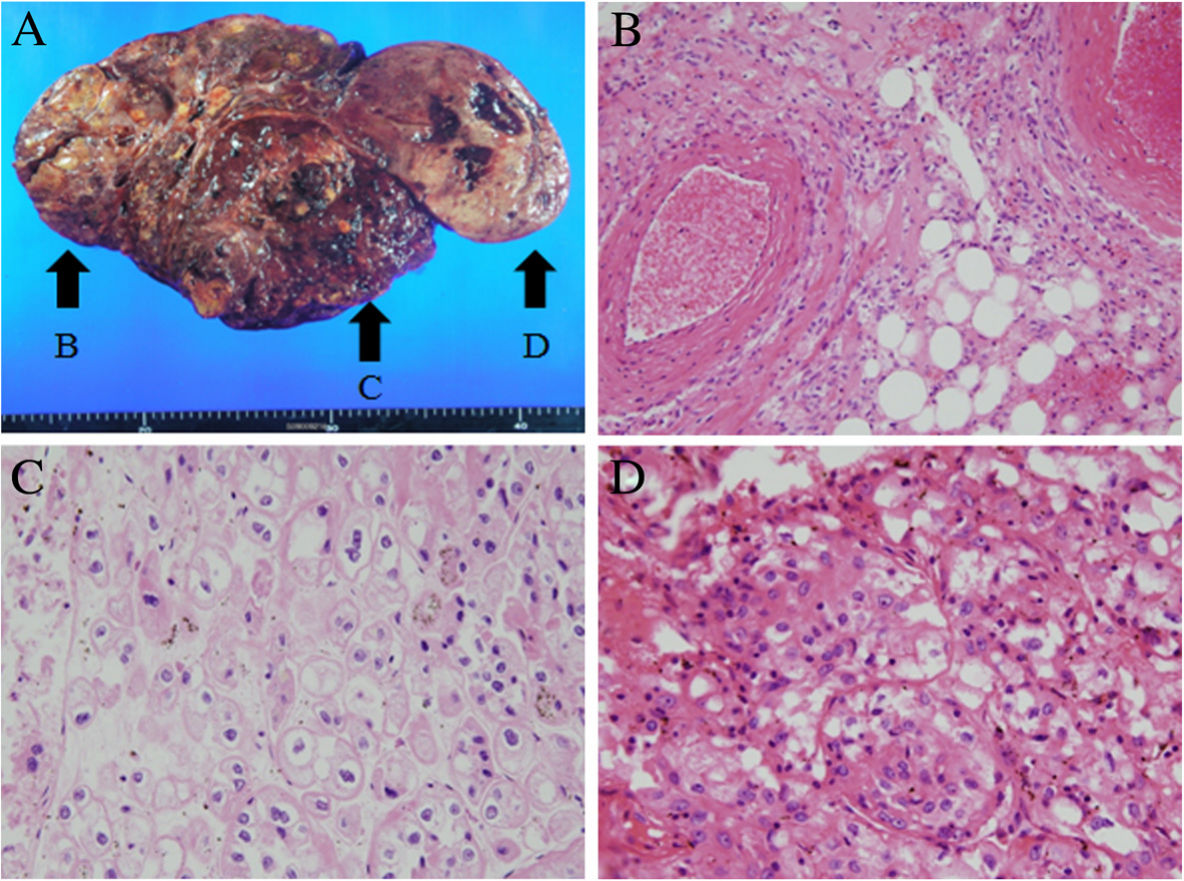
**Renal mass shows different pathologies. (A)** Photograph showing patient’s renal mass. Arrow B: AML; arrow C: chromophobe RCC; arrow D: clear cell carcinoma. **(B)** through **(D)** Histological tissue specimens showing AML **(B)**, chromophobe renal cell carcinoma **(C)** clear cell carcinoma **(D)**. Hematoxylin phloxine saffron stain; original magnification, 200×.

Abdominal sonography was performed to evaluate the patient’s fever and left flank pain, which revealed a newly developed mass alongside the AML in the left kidney. Because radiologic findings indicated the presence of renal clear cell carcinoma, we performed a radical left nephrectomy and diagnosed RCC on the basis of the histopathology. Pathologic examination revealed three kidney lesions (Figure 2A) of differing histologies (Figure 2A, arrow B: AML; arrow C: chromophobe RCC; arrow D: clear cell carcinoma). The final tumor node metastasis staging system classification was T3aN0M0, stage 3, Fuhrman nuclear grade 2.

In October 2010, approximately 17 months after the nephrectomy was performed, the patient experienced fever and left flank pain again. A follow-up CT scan revealed a heterogeneously enhanced mass at the nephrectomy site. A biopsy of the soft tissue in the left subphrenic area allowed us to confirm that this mass was recurrent RCC, and as a result a multidisciplinary team decided to administer systemic treatment for this unresectable and symptomatic disease. The first line of treatment included daily doses of sunitinib (50mg) for three months, which resulted in a partial response. However, recurrent grade 3 thrombocytopenia and stomatitis necessitated a 25% dose reduction. Additional dose reductions were necessary to address recurrent grade 2 hand-foot syndrome, whole-body folliculitis, grade 3 diarrhea and grade 2 hypothyroidism.

**Figure 3:**
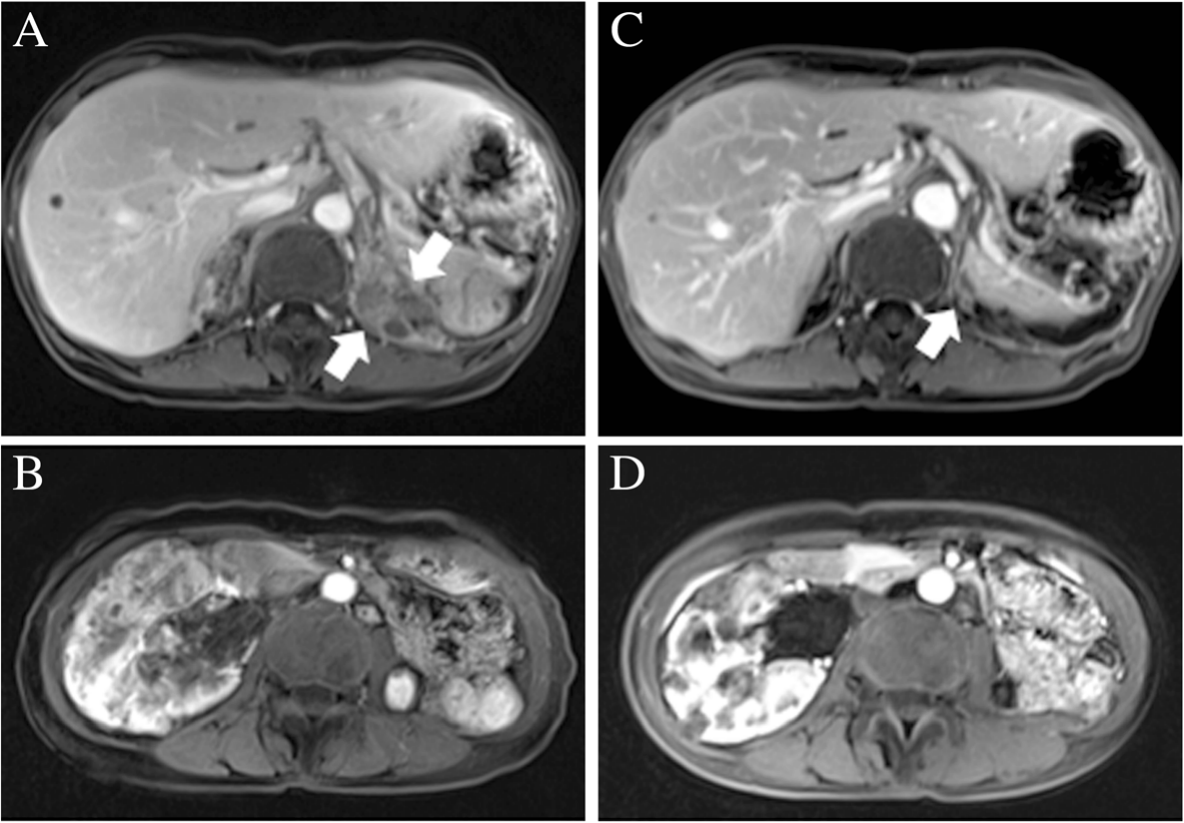
**Magnetic resonance images showing the treatment response of recurrent renal cell carcinoma and angiomyolipoma. (A)** Magnetic resonance imaging was used to produce this image showing recurrent renal cell carcinoma at the posterior aspect of left upper quadrant of abdomen (arrows). **(B)** Magnetic resonance imaging scan showing angiomyolipoma. **(C)** Magnetic resonance imaging scan showing partial response to everolimus treatment of renal cell carcinoma close to left diaphragm (arrow). **(D)** Imaging study showing regression of the patient’s angiomyolipoma.

After 13 months of sunitinib treatment, the lesion regrew and the patient’s pain recurred (Figure 3A and 3B). As second-line treatment, the patient was given oral everolimus (10mg/day) because it is approved for use in Korea for patients with recurrent RCC that is resistant to tyrosine kinase inhibitors (TKIs).

The patient reported complete resolution of pain after two months of everolimus treatment, and magnetic resonance imaging showed a partial response according to the Response Evaluation Criteria in Solid Tumours (RECIST 1.1) [2] (Figure 3C). Additionally, the patient’s facial fibroadenoma had remarkably regressed (Figure 1F), and, visualized on the follow-up images, the overall renal mass, including the AML lesion (Figure 3D), had decreased in size. Other asymptomatic benign lesions, including LAM (Figure 1G), cortical tubers (Figure 1H), subependymal nodules (Figure 1I) and SEGA (Figure 1J), had slightly improved. Adverse events were unremarkable with the exception of asymptomatic pneumonitis, which was observed in both basal lungs on the CT scan. As of December 2012, the patient’s treatment had achieved a partial response, and the patient was in stable condition and continued to receive treatment.

## Discussion

Elucidation of the roles of vascular endothelial growth factor (VEGF) and mTOR-containing complex 1 (mTORC1) in RCC has led to evaluation of the use of VEGF and mTOR inhibitors for the treatment of RCC. Increased understanding of the phosphatidylinositol 3-kinase (PI3K)/Akt/mTOR pathway has resulted in the development of various drugs that specifically target this signaling cascade [3]. Over the past decade, studies of these targeted agents have led to revolutionary changes in treatment options for patients with advanced RCC [1,4-8]. However, no validated predictive markers have been identified to guide decisions about which type of therapy should be chosen (VEGF-targeted or mTOR-targeted) [9,10]. Currently, the standard of care involves selection of the treatment regimen that is based solely on risk categories reflective of clinical parameters rather than on molecular pathogenesis.

Several known genes are associated with kidney cancer. Two of these genes, *TSC1* and *TSC2*, are downstream of *AMPK* and negatively regulate mTOR in response to cellular energy deficits. Germline mutations in either of these genes have been identified in patients with TSC, an autosomal dominant disorder associated with cutaneous angiofibromas, pulmonary lymphangiomyomatosis and renal tumors [1]. Renal lesions, including AML cysts and RCC, occur in 60% to 80% of TSC patients with TSC [11,12]. RCC has been reported in 1% to 4% of patients with TSC, which is a relatively low rate compared to that found in the whole general population. However, this type of cancer is typically diagnosed at a younger age in patients with TSC than in other patients with cancer. In a study group of patients with TSC, RCC developed at an average age of 28 years [11]. Washecka and Hanna also found in their review that 43% of TSC patients with RCC had bilateral disease. Although clear cell RCC is the predominant malignant histological subtype, TSC-related RCC is unusual in that it is histologically heterogeneous. Oncocytomas, as well as clear cell, papillary and chromophobe carcinoma subtypes, have been reported in patients with TSC [12,13]. Thus, the clinical course and prognosis of RCC associated with TSC are variable.

In most cases, TSC arises from mutations that inactivate the *TSC1* (hamartin) gene on chromosome 9 or the *TSC2* (tuberin) gene on chromosome 16 [14-16]. Either of these mutations results in constitutive mTOR expression and impaired tumor suppression in multiple tissues [17,18]. Consequently, the mTOR inhibitor rapamycin was evaluated in clinical, proof-of-principle trials. A total of 13 ongoing trials are being conducted to evaluate the efficacy of sirolimus, which is topical rapamycin, and everolimus, which is a rapamycin derivative, for treatment of various clinical TSC manifestations. Sirolimus treatment for either LAM of the lung [19] or AML of the kidney [19,20] has enhanced disease control in a trial setting.

Everolimus is currently licensed to treat patients who have advanced RCC and disease progression during or after treatment with VEGF-targeted therapy [6,21]. Before it was used as an antineoplastic agent, everolimus was approved as a prophylactic agent for organ rejection in adult patients receiving allogeneic renal or cardiac transplants.

Everolimus is a selective mTOR inhibitor that specifically targets mTORC1. The kinase activity of mTOR is activated mainly via the PI3K pathway. As mentioned above, several laboratory studies and case reports have suggested that mTOR inhibition leads to the shrinkage or stabilization of renal AML, LAM, facial angiofibroma and subependymal giant cell astrocytoma [22]. Recently, everolimus was approved in the European Union and the United States for the treatment of patients with SEGA associated with TSC. This approval was based on the results of a phase II study [23]. No evidence supports everolimus as first-line therapy over TKIs for patients with TSC as well as RCC. A case of a pediatric patient with RCC and TSC-related skin dermatofibrosis treated with sirolimus has been reported [24], but no data are available on everolimus as a treatment for RCC in adult patients with TSC.

## Conclusions

In this report, we describe the case of a patient who presented with RCC as a clinical manifestation of TSC. On the basis of the presence of TSC, the pathogenic mechanism that led to development of RCC in this patient was clearly defined. Despite our effective use of a TKI in accordance with treatment guidelines, the patient experienced a multitude of adverse events and her quality of life was significantly impaired during treatment. In contrast, second-line treatment with everolimus yielded significant clinical benefits evidenced by reductions in tumor size and other TSC manifestations, presumably because of inhibition of the most relevant RCC signaling target. Everolimus conferred these beneficial effects without detrimental adverse events and therefore did not compromise the patient’s quality of life. This observation provides a rationale for the use of everolimus as first-line treatment in this specific patient population to target the correct pathway involved in carcinogenesis.

## Consent

Written informed consent was obtained from the patient for publication of this case report and any accompanying images. A copy of the written consent is available for review by the Editor-in-Chief of this journal.

## Abbreviations

AML: Angiomyolipoma; CT: Computed tomography; LAM: Lymphangioleiomyomatosis; mTOR: Mammalian target of rapamycin; PI3K: Phosphatidylinositide 3-kinase; RCC: Renal cell carcinoma; SEGA: Subependymal giant cell astrocytoma; TKI: Tyrosine kinase inhibitor; TSC: Tuberous sclerosis complex; VEGF: Vascular endothelial growth factor.

## Competing interests

The authors declare that they have no competing interests.

## Authors’ contributions

KHP was directly involved in the overall care of the patient, served as the lead clinician, selected the images presented and obtained written consent from the patient. STK, DJS, CHK, SWS, YHK, and WYC were involved in the patient’s treatment and investigation of data. HSK was directly involved in the overall care of the patient and drafted the manuscript. All authors read and approved the final manuscript.

## References

[B1] Crino PB Nathanson KL Henske EP The tuberous sclerosis complex N Engl J Med 2006 355 1345 1356 10.1056/NEJMra055323 17005952

[B2] Eisenhauer EA Therasse P Bogaerts J Schwartz LH Sargent D Ford R Dancey J Arbuck S Gwyther S Mooney M Rubinstein L Shankar L Dodd L Kaplan R Lacombe D Verweij J New response evaluation criteria in solid tumours: revised RECIST guideline (version 1.1) Eur J Cancer 2009 45 228 247 10.1016/j.ejca.2008.10.026 19097774

[B3] Linehan WM Srinivasan R Schmidt LS The genetic basis of kidney cancer: a metabolic disease Nat Rev Urol 2010 7 277 285 10.1038/nrurol.2010.47 20448661 PMC2929006

[B4] Escudier B Bellmunt J Négrier S Bajetta E Melichar B Bracarda S Ravaud A Golding S Jethwa S Sneller V Phase III trial of bevacizumab plus interferon α-2a in patients with metastatic renal cell carcinoma (AVOREN): final analysis of overall survival J Clin Oncol 2010 28 2144 2150 10.1200/JCO.2009.26.7849 20368553

[B5] Escudier B Eisen T Stadler WM Szczylik C Oudard S Staehler M Negrier S Chevreau C Desai AA Rolland F Demkow T Hutson TE Gore M Anderson S Hofilena G Shan M Pena C Lathia C Bukowski RM Sorafenib for treatment of renal cell carcinoma: final efficacy and safety results of the phase III treatment approaches in renal cancer global evaluation trial J Clin Oncol 2009 27 3312 3318 10.1200/JCO.2008.19.5511 19451442

[B6] Motzer RJ Escudier B Oudard S Hutson TE Porta C Bracarda S Grünwald V Thompson JA Figlin RA Hollaender N Urbanowitz G Berg WJ Kay A Lebwohl D Ravaud A RECORD-1 Study Group Efficacy of everolimus in advanced renal cell carcinoma: a double-blind, randomised, placebo-controlled phase III trial Lancet 2008 372 449 456 10.1016/S0140-6736(08)61039-9 18653228

[B7] Motzer RJ Hutson TE Tomczak P Michaelson MD Bukowski RM Rixe O Oudard S Negrier S Szczylik C Kim ST Chen I Bycott PW Baum CM Figlin RA Sunitinib versus interferon α in metastatic renal-cell carcinoma N Engl J Med 2007 356 115 124 10.1056/NEJMoa065044 17215529

[B8] Sternberg CN Davis ID Mardiak J Szczylik C Lee E Wagstaff J Barrios CH Salman P Gladkov OA Kavina A Zarbá JJ Chen M McCann L Pandite L Roychowdhury DF Hawkins RE Pazopanib in locally advanced or metastatic renal cell carcinoma: results of a randomized phase III trial J Clin Oncol 2010 28 1061 1068 10.1200/JCO.2009.23.9764 20100962

[B9] Armstrong AJ George DJ Halabi S Serum lactate dehydrogenase predicts for overall survival benefit in patients with metastatic renal cell carcinoma treated with inhibition of mammalian target of rapamycin J Clin Oncol 2012 30 3402 3407 10.1200/JCO.2011.40.9631 22891270

[B10] Atkins M Regan M McDermott D Mier J Stanbridge E Youmans A Febbo P Upton M Lechpammer M Signoretti S Carbonic anhydrase IX expression predicts outcome of interleukin 2 therapy for renal cancer Clin Cancer Res 2005 11 3714 3721 10.1158/1078-0432.CCR-04-2019 15897568

[B11] Washecka R Hanna M Malignant renal tumors in tuberous sclerosis Urology 1991 37 340 343 10.1016/0090-4295(91)80261-5 2014599

[B12] Bjornsson J Short MP Kwiatkowski DJ Henske EP Tuberous sclerosis-associated renal cell carcinoma: clinical, pathological, and genetic features Am J Pathol 1996 149 1201 1208 8863669 PMC1865172

[B13] Robertson FM Cendron M Klauber GT Harris BH Renal cell carcinoma in association with tuberous sclerosis in children J Pediatr Surg 1996 31 729 730 10.1016/S0022-3468(96)90689-2 8861495

[B14] Nellist M Janssen B Brook-Carter PT Hesseling-Janssen ALW Maheshwar MM Verhoef S Van den Ouweland AMW Lindhout D Eussen B Cordeiro I Santos H Halley DJJ Sampson JR Ward CJ Peral B Thomas S Hughes J Harris PC Roelfsema JH Saris JJ Spruit L Peters DJM Dauwerse JG Breuning MH European Chromosome 16 Tuberous Sclerosis Consortium Identification and characterization of the tuberous sclerosis gene on chromosome 16 Cell 1993 75 1305 13158269512

[B15] Fryer AE Chalmers A Connor JM Fraser I Povey S Yates AD Yates JR Osborne JP Evidence that the gene for tuberous sclerosis is on chromosome 9 Lancet 1987 1 659 661 2882085 10.1016/s0140-6736(87)90416-8

[B16] Kandt RS Haines JL Smith M Northrup H Gardner RJ Short MP Dumars K Roach ES Steingold S Wall S Blanton SH Flodman P Kwiatkowski DJ Jewell A Weber JL Roses AD Pericak-Vance MA Linkage of an important gene locus for tuberous sclerosis to a chromosome 16 marker for polycystic kidney disease Nat Genet 1992 2 37 41 10.1038/ng0992-37 1303246

[B17] Huang J Manning BD The TSC1–TSC2 complex: a molecular switchboard controlling cell growth Biochem J 2008 412 179 190 10.1042/BJ20080281 18466115 PMC2735030

[B18] Cheadle JP Reeve MP Sampson JR Kwiatkowski DJ Molecular genetic advances in tuberous sclerosis Hum Genet 2000 107 97 114 10.1007/s004390000348 11030407

[B19] Bissler JJ McCormack FX Young LR Elwing JM Chuck G Leonard JM Schmithorst VJ Laor T Brody AS Bean J Sheila S Franz DN Sirolimus for angiomyolipoma in tuberous sclerosis complex or lymphangioleiomyomatosis N Engl J Med 2008 358 140 151 10.1056/NEJMoa063564 18184959 PMC3398441

[B20] Dabora SL Franz DN Ashwal S Sagalowsky A DiMario FJ Jr Miles D Cutler D Krueger D Uppot RN Rabenou R Camposano S Paolini J Fennessy F Lee N Woodrum C Manola J Garber J Thiele EA Multicenter phase 2 trial of sirolimus for tuberous sclerosis: kidney angiomyolipomas and other tumors regress and VEGF-D levels decrease PLoS One 2011 6 e23379 10.1371/journal.pone.0023379 21915260 PMC3167813

[B21] Motzer RJ Escudier B Oudard S Hutson TE Porta C Bracarda S Grünwald V Thompson JA Figlin RA Hollaender N Kay A Ravaud A Phase 3 trial of everolimus for metastatic renal cell carcinoma: final results and analysis of prognostic factors Cancer 2010 116 4256 4265 10.1002/cncr.25219 20549832

[B22] Govindarajan B Willoughby L Band H Curatolo AS Veledar E Chen S Bonner MY Abel MG Moses MA Arbiser JL Cooperative benefit for the combination of rapamycin and imatinib in tuberous sclerosis complex neoplasia Vasc Cell 2012 4 11 10.1186/2045-824X-4-11 22765013 PMC3464934

[B23] Krueger DA Care MM Holland K Agricola K Tudor C Mangeshkar P Wilson KA Byars A Sahmoud T Franz DN Everolimus for subependymal giant-cell astrocytomas in tuberous sclerosis N Engl J Med 2010 363 1801 1811 10.1056/NEJMoa1001671 21047224

[B24] Pressey JG Wright JM Geller JI Joseph DB Pressey CS Kelly DR Sirolimus therapy for fibromatosis and multifocal renal cell carcinoma in a child with tuberous sclerosis complex Pediatr Blood Cancer 2010 54 1035 1037 20108343 10.1002/pbc.22401

